# An expertized grapevine disease image database including five grape varieties focused on Flavescence dorée and its confounding diseases, biotic and abiotic stresses

**DOI:** 10.1016/j.dib.2023.109230

**Published:** 2023-05-12

**Authors:** Malo Tardif, Ahmed Amri, Aymeric Deshayes, Marc Greven, Barna Keresztes, Gaël Fontaine, Laetitia Sicaud, Laetitia Paulhac, Sophie Bentejac, Jean-Pierre Da Costa

**Affiliations:** a Univ. Bordeaux, CNRS, Bordeaux INP, IMS, UMR 5218, Talence F-33400, France; b INRAE, EGFV, Villenave d'Ornon F-33140, France; c Bordeaux Sciences Agro, Gradignan F-33175, France; d Bureau National Interprofessionnel du Cognac, Cognac FF-16100, France; e FREDON Nouvelle-Aquitaine, Cognac F-16100, France; f GDON de Bordeaux, Beychac et Caillau F-33750, France

**Keywords:** Grapevine, Grapevine disease, Esca, Proximal sensing, RGB images, Detection, Classification

## Abstract

The grapevine is vulnerable to diseases, deficiencies, and pests, leading to significant yield losses. Current disease controls involve monitoring and spraying phytosanitary products at the vineyard block scale. However, automatic detection of disease symptoms could reduce the use of these products and treat diseases before they spread. Flavescence dorée (FD), a highly infectious disease that causes significant yield losses, is only diagnosed by identifying symptoms on three grapevine organs: leaf, shoot, and bunch. Its diagnosis is carried out by scouting experts, as many other diseases and stresses, either biotic or abiotic, imply similar symptoms (but not all at the same time). These experts need a decision support tool to improve their scouting efficiency.

To address this, a dataset of 1483 RGB images of grapevines affected by various diseases and stresses, including FD, was acquired by proximal sensing. The images were taken in the field at a distance of 1-2 meters to capture entire grapevines and an industrial flash was ensuring a constant luminance on the images regardless of the environmental circumstances. Images of 5 grape varieties (Cabernet sauvignon, Cabernet franc, Merlot, Ugni blanc and Sauvignon blanc) were acquired during 2 years (2020 and 2021).

Two types of annotations were made: expert diagnosis at the grapevine scale in the field and symptom annotations at the leaf, shoot, and bunch levels on computer. On 744 images, the leaves were annotated and divided into three classes: ‘FD symptomatic leaves’, ‘Esca symptomatic leaves’, and ‘Confounding leaves’. Symptomatic bunches and shoots were, in addition of leaves, annotated on 110 images using bounding boxes and broken lines, respectively. Additionally, 128 segmentation masks were created to allow the detection of the symptomatic shoots and bunches by segmentation algorithms and compare the results to those of the detection algorithms.

**Specifications Table**

**Table:** 

Subject	Agronomy and Crop Science
Specific subject area	The specific subject area is the automatic detection of grapevine diseases. The dataset focuses on images of one grapevine disease called Flavescence dorée, very closely monitored in Europe, and its confounding factors.
Type of data	Image Annotation files (.json)
How the data were acquired	Images were acquired from the rows using an acquisition system mounted on a customized wheelbarrow. It is composed of a 5 Mpx industrial Basler Ace (acA2440-20gc GigE, Basler AG, Ahrensburg, Germany) global shutter RGB camera with a 6 mm focal length (70° horizontal field of view) lens. It also includes a high-power Phoxene Sx-3 xenon flash. For each vine image, the experts established what the grapevine suffered from, creating a first annotation at the image scale. The same experts made annotations on the images themselves, either with bounding boxes (made with the “labelme” software [1]) or dense region masks (performed with the “Gimp” software [2]).
Data format	Raw Analyzed Annotated
Description of data collection	Images have been acquired of 5 grape varieties (Cabernet sauvignon, Cabernet franc, Merlot, Ugni blanc, Sauvignon blanc), during two years (2020, 2021) and on 14 vineyard blocks. To be photographed a grapevine had to be: • affected by FD. • affected by Esca. • presenting symptoms similar to those of FD or Esca.
Data source location	All the data were acquired in the Nouvelle Aquitaine region, in France.In 2020: • City/Town/Region: Réparsac, Charente Latitude and longitude: ○ Plot 1: 45.7406102, -0.2293657 ○ Plot 2: 45.7414515, -0.2271748 ○ Plot 3: 45.7409606, -0.2255990 ○ Plot 4: 45.7405824, -0.2293786
	• City/Town/Region: Saint-Sève, Gironde Latitude and longitude: ○ Plot 1: 44.6126886, -0.0379963 ○ Plot 2: 44.6102027, -0.0389426 ○ Plot 3: 44.6255832, -0.3070293 • City/Town/Region: Louviac, Gironde, Latitude and longitude: ○ Plot 1: 44.6071638, -0.2460330 • City/Town/Region: Semens, Gironde, Latitude and longitude: ○ Plot 1: 44.6058326, -0.2424245 ○ Plot 2: 44.5750228, -0.2468006 • City/Town/Region: Saint-Maixant, Gironde Latitude and longitude: ○ Plot 1: 44.5750412, -0.2467987 In 2021: • City/Town/Region: Réparsac, Charente Latitude and longitude: ○ Plot 1: 45.7391416, -0.2286879 • City/Town/Region: Saint-Laurent, Charente, Latitude and longitude: ○ Plot 1: 45.3702535, 0.0334376 • City/Town/Region: Langoiran, Gironde Latitude and longitude: ○ Plot 1: 44.6992974, -0.3924154 • City/Town/Region: Rions, Gironde Latitude and longitude: ○ Plot 1: 44.6704526, -0.3561660 ○ Plot 2: 44.6726088, -0.3610193 • City/Town/Region: Saint-Martin, Gironde Latitude and longitude: ○ Plot 1: 44.5712274, -0.1697558
Data accessibility	Repository name: Mendeley Data Direct URL to data: https://data.mendeley.com/datasets/3dr9r3w3jn/2
Related research article	Tardif, M., Amri, A., Keresztes, B., Deshayes, A., Martin, D., Greven, M., & Da Costa, J.-P. (2022). Two-stage automatic diagnosis of Flavescence Dorée based on proximal imaging and artificial intelligence: a multi-year and multi-variety experimental study. OENO One, 56(3), 371–384. https://doi.org/10.20870/oeno-one.2022.56.3.5460

## Value of the Data

• This dataset is very complete as it covers more than 1400 images of 5 grape varieties acquired in 14 different blocks annotated at the image scale. More than 800 of these images are annotated at the symptom scale. These images can be very useful to train and test many algorithms (deep or non-deep learning algorithms) to automatically diagnose grapevine diseases. In the related research article, 2 segmentation algorithms (ResUnet, structure tensor) and one deep detection algorithm (YOLOV4-tiny) have been trained and tested on this dataset to automatically detect the symptoms of FD. The annotations available, realized by experts, allow a diagnosis at the symptom scale and at the grapevine scale. Using these data can save a lot of acquisition and annotation time.
• All the researchers and professionals whose activities are related to the monitoring of phytopathologies, can benefit from these data. However, the data can also benefit other precision viticulture applications. The annotations were made by experts so they can benefit all those who don't have the possibility to get certified annotated images.
• The data can be used by researchers or developers of computer vision and machine learning. In this case, the data are of particular interest because they contain annotations of different kinds: at the image scale and at the symptom scale. And in this last case, they have taken different forms: boxes, broken lines and regions.
• These data can be used to create a first grapevine diseases dataset or to complete an existing one. The annotations at the symptom scale can be used to develop or improve symptom detection algorithms, while the annotations at the grapevine scale can be used to develop or improve an automatic diagnostic tool of grapevine diseases.

## 1 Objective

This dataset was created to be very challenging for the automatic diagnosis of FD as all images in this dataset display grapevines affected by some stress factor, biotic or abiotic, showing symptoms similar to those of FD. This dataset contains images of 5 grape varieties acquired in 14 blocks to present as many variabilities in the symptom expressions as possible.

The idea for this particular study comes from an intensive literature review. Many studies reached very accurate results in the automatic diagnosis of FD with images acquired by proximal sensing [3] or UAV [4], [5], [6], [7]. However, these results have been obtained for the discrimination between healthy grapevines and grapevines affected by FD, on a single variety, or several varieties but with very few data.

Where field experts manage to do identify the several concomitant symptoms to detect the presence of FD without any problem, the bibliographic study has shown how difficult this is for machine learning and deep learning approaches. We therefore wanted to go beyond simple annotation at the plant level by producing annotations for all visible symptoms, in order to develop algorithmic approaches based on the explicit association of symptoms [8].

## 2 Data Description

Flavescence dorée (FD) is a disease that is closely monitored in Europe and has been classified as a quarantine disease at the European level since 1993. The main vector of this disease is the leafhopper *Scaphoideus titanus* Ball, which transmits the phytoplasma “*Candidatus vitis*” during phloem feeding. Without control measures, the disease can spread rapidly and affect the entire vineyard in a few years, causing significant economic consequences for winegrowers. Expertise is necessary to diagnose FD, as there are many phytosanitary diseases with symptoms similar to those of FD. In order to distinguish FD from its confounding diseases, experts not only rely on the leaf symptoms (the most visible and confounding symptoms), but on the combination of the 3 symptoms of FD on the same vine. Symptoms of FD appear on three organs of the affected vine: the leaves, shoots, and bunches. For red grape varieties, the leaves turn red, while for white varieties, they turn yellow, with a possibility of rolling. Symptomatic shoots are identified by a lack of lignification, meaning they do not undergo the natural browning process that makes them resistant to frost. At the bunch level, the berries become wilted at a very early stage and the inflorescences dry out.

**Fig. 1:**
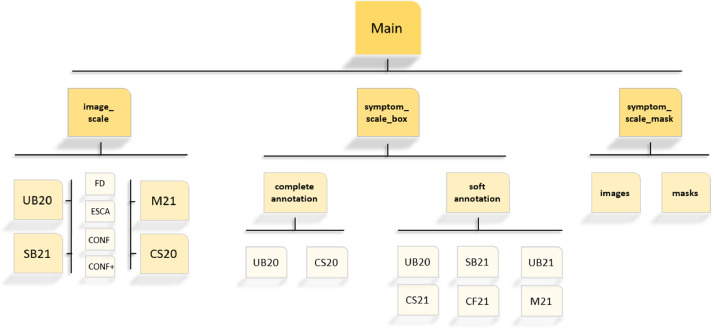
Representation of how the data are distributed in the available dataset.

This dataset contains 1483 images and their different annotations (Table 1). The images and annotation files in this dataset are divided into 3 main folders, without overlap (Fig. 1):

• The first folder is called ‘image_scale’ and contains the annotation at the image scale (i.e at the grapevine scale) of all the images not annotated at the symptom scale. The images are first classified into folders according to grape variety and acquisition year. They are then sorted depending on which disease the photographed grapevine was suffering. This sorting was done during the acquisition with the expert, who indicated the disease presence on the grapevine. Four folders are used: either it is a grapevine affected by FD (‘FD’ folder), by Esca (‘ESCA’ folder), by a confounding disease (‘CONF’ folder) or by a very confounding disease (‘CONF+’ folder). The classification between these two last classes is an arbitrary choice. When looking at the images, those with visual symptoms (especially those on leaves) very similar to those of FD were put in the ‘CONF+’ class. The aim of this split was to investigate the accuracy of the algorithms on the most difficult cases to treat. More information about this classification is available in [8]. These annotations can be used for classification algorithms.
• The second folder, called ‘symptom_scale_box’, contains images and their symptom annotations with bounding boxes and line strips created with the Labelme software. The annotation files were saved in the ‘.json’ format. Each annotated symptom is described in these files by its label (the class of the symptom), the coordinates of its bounding box or line strip ([[xmin, ymin], [xmax, ymax]]) and its shape type (bounding box or line strip). These annotations can be used for detection algorithms. There are 2 types of annotations in this folder, called “soft annotation” and “complete annotation”.
  ○ The ‘soft annotation’ consists of bounding boxes of the classes: “FD leaf” (symptomatic leaf of FD), “ESCA leaf” (symptomatic leaf of ESCA) and “confounding leaf” (leaves that are visually different from healthy leaves). These data can only be used to automatically detect the symptomatic leaves of FD, ESCA and the confounding leaves.
  ○ The ‘complete annotation’ also contains leaf bounding boxes of the 3 above classes but is completed by bounding boxes of the class “symptomatic bunch” (dried out bunches) and by line strips of the class “symptomatic shoot” (unlignified shoots). This can be used to automatically detect the symptomatic leaves of FD and of ESCA, the confounding leaves, as well as the symptomatic shoots and bunches of FD.

All these images and annotation files are sorted into folders depending on the grape variety and the acquisition year. An annotation file corresponds to an image if they are in the same folder and have the same name, except from the extension (‘.jpg’ for the images, ‘.json’ for the annotation files).

Among the symptoms of FD, the most complicated to differentiate from the confounding symptoms, are those on the leaves. Because we wanted as many annotated leaves as possible and the ‘complete annotation’ files were too time consuming to create for the experts, it was decided to annotate only the leaves in a ‘soft annotation’. With regard to the shoots and bunches, it was decided to annotate them, based on the expert annotations of these symptoms, by segmentation masks to allow the comparison of different algorithms for their detection.

The third folder, ‘symptom_scale_mask’, contains 128 images and their associated segmentation masks. The images are in the folder ‘images’ and the masks in the folder ‘mask’. A segmentation mask corresponds to an image if it has the same name, except from the extension (‘.jpg’ for the images, ‘.png’ for the masks). Four classes of pixels have been created using the GIMP software: either it was a pixel of a symptomatic shoot (RGB values [100,0,0]), symptomatic bunch ([0,100,0]), healthy bunch ([0,0,100]) or a pixel of all the rest ([0,0,0]). These annotations can be used for segmentation algorithms.

Table 1. summarizes the data acquisitions and annotations performed in 2020 and 2021. Images and annotations are classified depending on the grape variety and the acquisition year. The names of the corresponding folders in the dataset are indicated. There were only annotations with segmentation masks in 2020.

Fig. 1. allows a better comprehension of how the data are distributed in the available dataset. There are three main folders, one for each annotation type. In the “image_scale” folder (which means the annotations at the grapevine scale), data are first divided into folders depending on the grape variety and the acquisition year, and then distributed depending on the disease at the grapevine scale. Then the “symptom_scale_box” folder contains the images and their associated annotations at the symptom scale created with the labelme software (.json files). They are first divided into 2 folders depending on the type of annotation (soft/complete) and then by grape variety and acquisition year. Finally, the “symptom_scale_mask” folder contains the images and their associated segmentation masks realized with the GIMP software.

**Table 1:** Resume of the number of acquired and annotated images per grape variety and acquisition year.

Grape variety *(acquisition year; folder name)*	Cabernet sauvignon *(2020; CS20)*	Ugni blanc *(2020; UB20)*	Cabernet sauvignon *(2021; CS21)*	Merlot *(2021; M21)*	Cabernet franc *(2021; CF21)*	Ugni blanc *(2021; UB21)*	Sauvignon blanc *(2021; SB21)*
**Total of images**	**405**	**463**	**116**	**98**	**86**	**161**	**154**
**No. images annotated at the image scale**	**260**	**192**	**0**	**55**	**0**	**0**	**104**

Of which FD	72	83		32			41
Of which Esca	45	13		5			44
Of which CONF	87	88		7			17
Of which CONF+	56	8		11			2

**No. images annotated by bounding boxes**	**67**	**221**	**116**	**43**	**86**	**161**	**50**
**No. images annotated by segmentation masks**	**78**	**50**					

**Fig. 2:**
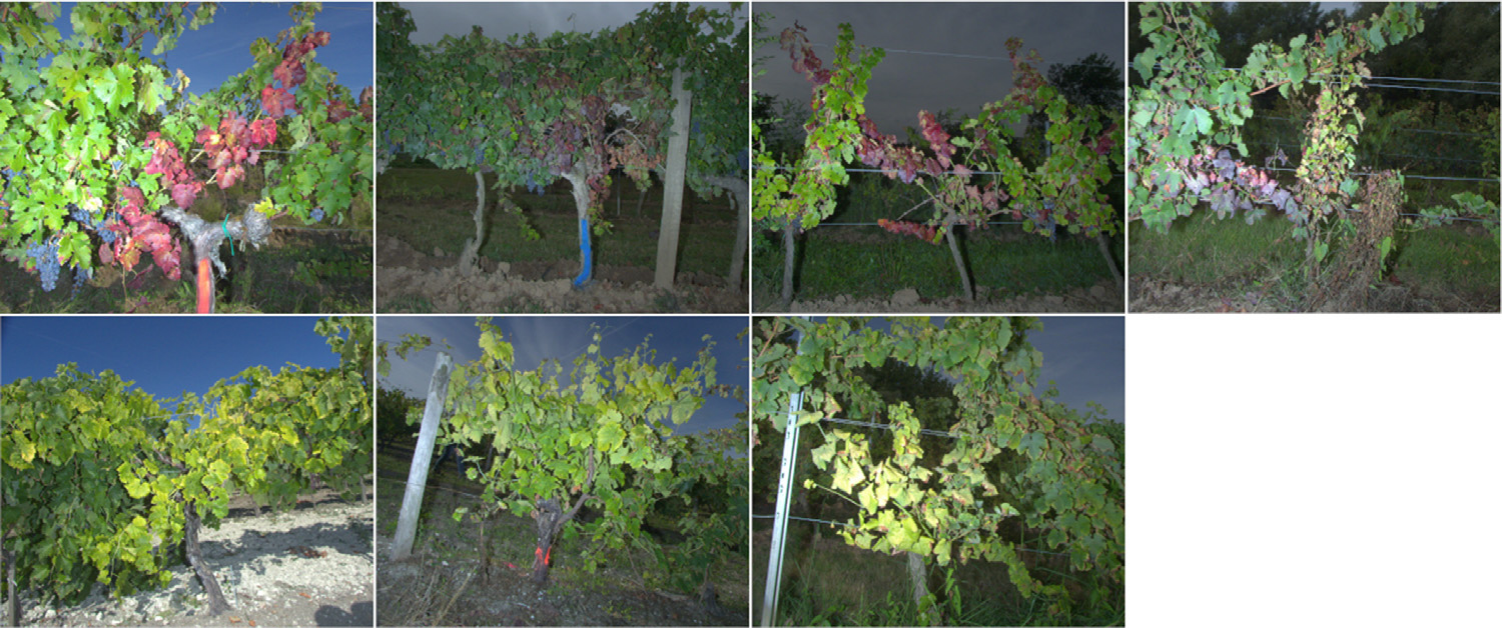
Symptomatic grapevine suffering from FD of each dataset. From top to bottom, left to right: image of the dataset CS20, CS21, CF21, M21, UB20, UB21, SB21.

Fig. 2 shows images of symptomatic grapevines affected by FD for several grape varieties and acquisition years. It can be noticed that acquisition conditions and symptom expressions may vary between plots and varieties.

**Fig. 3:**
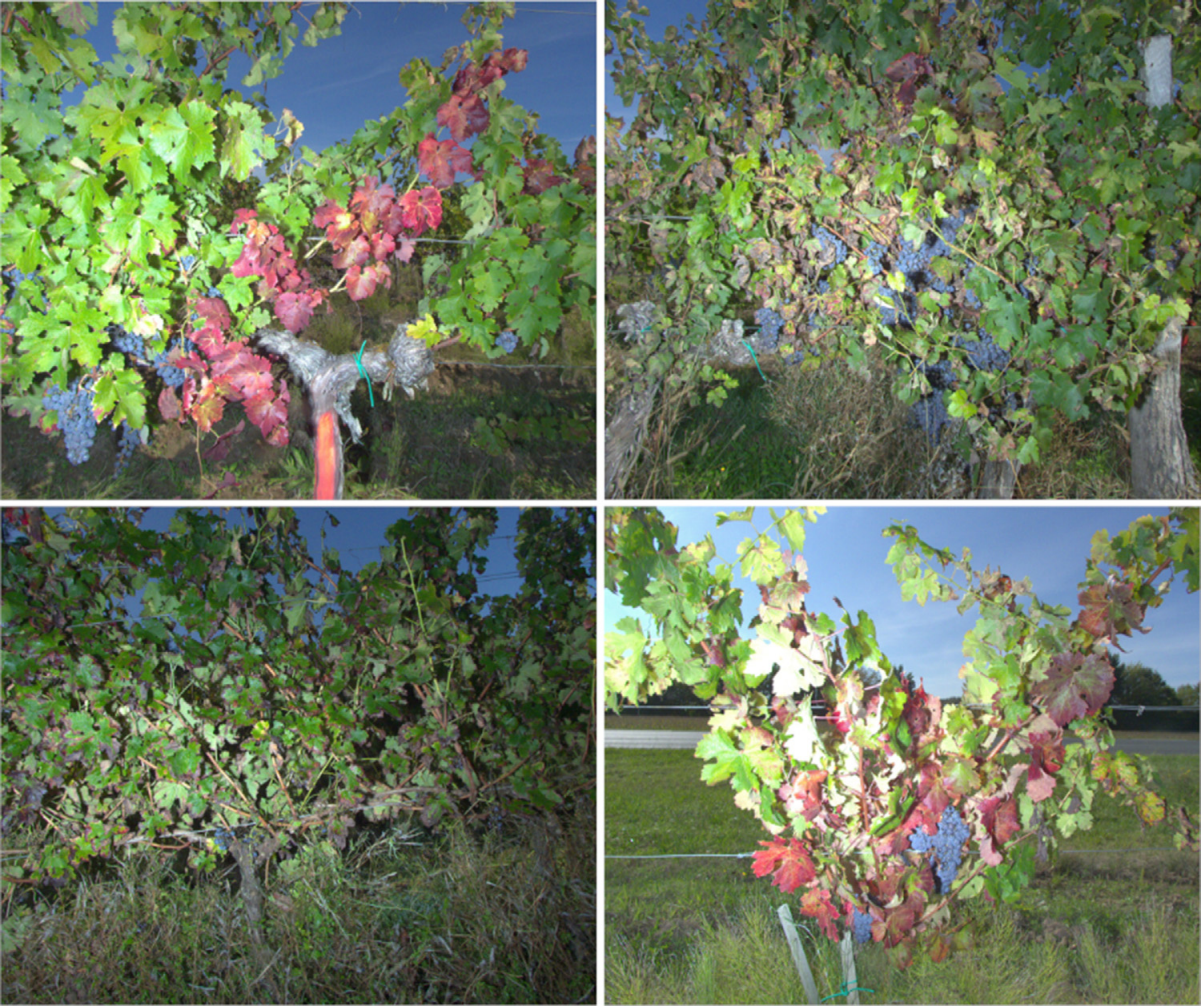
Example of images of the classes (from top to bottom; left to right) "FD", "ESCA", "CONF" and "CONF+" of the CS20 dataset.

Fig. 3 shows images of the classes ‘FD’, ‘ESCA’, ‘CONF’ and ‘CONF+’ taken on Cabernet Sauvignon grapevines photographed in 2020. One can see the very close similarity of the visual symptoms between the image of the 'FD' class and the one of the 'CONF+' class.

**Fig. 4:**
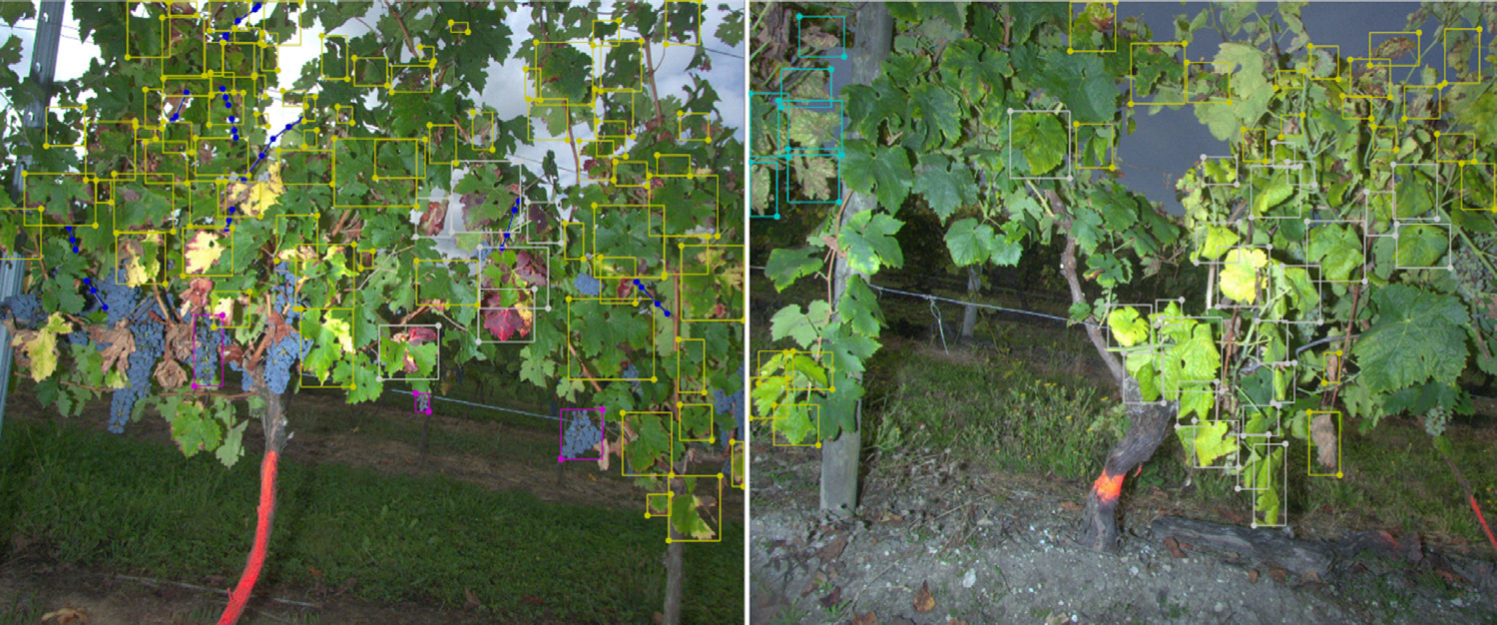
An example of complete (left image) and soft annotation (right image) by bounding boxes and broken lines. In grey: Symptomatic leaves of FD. In yellow: confounding leaves. In purple: Symptomatic bunches. In dark blue: symptomatic shoots. In light blue: symptomatic leaves of ESCA.

Fig. 4 shows the two types of symptom annotations performed by experts. These images are screenshots of the labelme software. The image at the left shows a “complete” annotation of an image in the ‘CS20’ folder while the image at the right shows a “soft annotation” of an image in the ‘UB20’ folder.

**Fig. 5:**
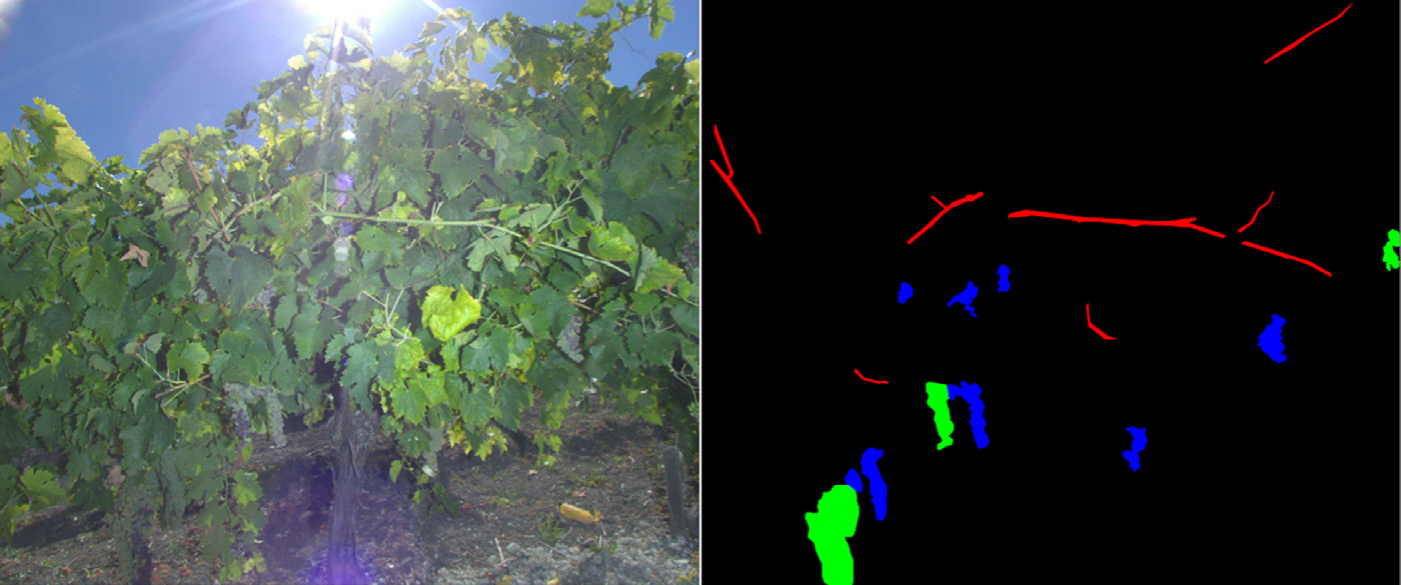
Example of an image and its associated segmentation mask. In red: pixels of symptomatic shoots. In green: pixels of symptomatic bunches. In blue: pixels of healthy bunches. In black: pixels of everything else.

Fig. 5 shows an image of the ‘CS20’ folder and its associated segmentation mask created with the GIMP software using the ‘Free Select Tool’.

## 3 Experimental Design, Materials and Methods

The images were acquired directly in the field with the acquisition system mounted on a wheelbarrow. It is composed of a 5 Mpx industrial Basler Ace (acA2440-20gc GigE, Basler AG, Ahrensburg, Germany) global shutter RGB camera with a 6mm focal length (70° horizontal field of view) lens.

It also includes a high-power Phoxene Sx-3 xenon flash used with a short exposure time (250 µs) to have a controlled lighting and constant luminance on each image whatever the environmental circumstances. An on-board embedded computer controls the camera and stores of the images. A 12V battery powers the entire system. A more detailed description of the acquisition device is available in [9].

In two years, we went with scouting experts to 14 blocks, planted with 5 different grape varieties, identified as containing many cases of FD. We acquired the images in September and October, just before the harvest in France, when the symptoms are best expressed. Images were taken at a distance between 1 and 2 meters depending on the size of the rows in order to capture the entire grapevine (Fig. 2).

The acquisitions were focused on one disease called Flavescence dorée and its confounding diseases. We divided the dataset into 4 classes depending on the disease symptoms present on the images: ‘FD’, ‘ESCA’, ‘CONF’, ‘CONF+’ (Fig. 3).

Once the images were acquired, the same scouting experts were asked to annotate the images on a computer screen, using the label me software (Fig. 4). They created bounding boxes around the objects of interest: the symptomatic leaves of FD, ESCA, the confounding leaves (leaves visually different from healthy leaves) and the symptomatic bunches of FD. Because of the shape of the symptomatic shoots, bounding boxes were not appropriate to annotate them. Hence the shoots were annotated by using broken lines. The annotation files were finally saved in the ‘.json’ format.

Segmentation masks were also created for the symptomatic shoots, bunches and the healthy bunches (Fig. 5). They were created with the GIMP software using the ‘Free select Tool’ and saved in the ‘.png’ format.

## Ethics Statements

The data presented in this study did not involve using human or animal subjects or social media platforms. Therefore, no ethical statements as per the journal policy were required for the data.

## CRediT Author Statement

**Malo Tardif:** Writing – original draft, Investigation, Software, Methodology, Validation, Data Curation; **Ahmed Amri:** Investigation, Data Curation, Software; **Aymeric Deshayes:** Writing – original draft, Investigation, Data Curation; **Marc Greven:** Writing – review & editing, Supervision, Methodology; **Barna Keresztes:** Investigation, Camera and Software development, Resources; **Gaël Fontaine:** Investigation, Resources; **Laetitia Sicaud:** Investigation, Supervision, Validation, Data Curation **Laetitia Paulhac:** Investigation, Supervision, Validation, Data Curation; **Sophie Bentejac:** Investigation, Supervision, Validation, Data Curation; **Jean-Pierre Da Costa:** Writing – review & editing, Supervision, Project Administration, Methodology.

## Declaration of Competing Interest

The authors declare that they have no known competing financial interests or personal relationships that could have appeared to influence the work reported in this paper.

## Data Availability

An expertized grapevine disease image database focused on Flavescence dorée and its confounding diseases (Original data) (Mendeley Data).
